# Routine Removal of Syndesmotic Screws After Tibiofibular Syndesmosis Fixation Does Not Affect Patient Function and Is Associated with a Higher Risk of Postoperative Complications

**DOI:** 10.3390/jcm14103276

**Published:** 2025-05-08

**Authors:** Błażej Grzegorz Wójtowicz, Katarzyna Chawrylak, Jędrzej Lesman, Hubert Makowski, Kacper Kuczyński, Michał Maciejowski, Antoni Raciborski-Król, Marcin Domżalski

**Affiliations:** 1 Department of Orthopedics and Trauma, Medical University of Lodz, Veteran’s Memorial Hospital, Zeromskiego 113 St., 90-549 Lodz, Poland; jedreklesman@yahoo.pl (J.L.); marcin.domzalski@umed.lodz.pl (M.D.); 2 Department of Surgical Oncology, Medical University of Lublin, Radziwiłłowska 13 St., 20-080 Lublin, Poland; katchawrylak@gmail.com; 3 Medical University of Lodz, Al. Tadeusza Kościuszki 4, 90-419 Lodz, Poland; hubert.makowski@stud.umed.lodz.pl (H.M.); kacper.kuczynski@stud.umed.lodz.pl (K.K.); michal.maciejowski@stud.umed.lodz.pl (M.M.); antoni.raciborski-krol@student.umed.lodz.pl (A.R.-K.)

**Keywords:** syndesmosis fixation, routine removal, ankle fractures, OMAS, syndesmotic injury, patient-reported outcomes

## Abstract

**Background:** Syndesmotic fixation is a standard treatment for tibiofibular syndesmosis injury, especially in ankle fractures, but the necessity of routine screw removal remains debated. This study evaluates the impact of routine removal on functional outcomes, complication rates, and recovery, aiming to clarify its clinical relevance. **Methods:** This retrospective observational study included 330 patients treated surgically for tibiofibular syndesmosis injuries at a single institution from 2016 to 2024. Patients were categorized into three groups: no removal, routine removal, and removal for clinical indications. Functional outcomes were assessed using the Olerud–Molander Ankle Score (OMAS), and complications, including infections and prolonged pain, were recorded. Statistical analyses evaluated differences between groups. **Results:** Of the 170 patients who completed follow-up, no significant differences in OMASs were found between the groups (*p* = 0.646). Complications were more frequent in reoperated patients (9% vs. 2% for non-reoperated), but routine removal did not improve functional outcomes. Younger patients showed better OMASs, highlighting age as a key factor in recovery. Fixation and fracture types had no significant impact on outcomes. **Conclusions:** Routine syndesmosis screw removal offers no significant functional benefits, as demonstrated by comparable OMASs across groups (median OMAS: 85 for non-reoperated, 80 for routinely reoperated, and 80 for indication-based reoperated patients). However, routine removal is associated with a higher complication rate. A selective approach focusing on clinical indications is recommended to minimize unnecessary surgeries and optimize patient outcomes. Future research should focus on redefining evidence-based management strategies for syndesmotic fixation.

## 1. Introduction

Acute ankle sprains are a frequent injury that significantly affects both athletic performance and everyday activities [1].

Certain populations are more susceptible to ankle sprains. Females experience a higher incidence rate (13.6 per 1000 exposures) than males (6.9 per 1000 exposures), and children are more frequently affected than adults [1,2].

Ankle fractures make up 10% of all fractures, with a rate of 184 per 100,000 person-years in northern Europe. The actual rate is likely higher due to underreporting [3,4]. These fractures are the second most common lower limb fractures after hip fractures [5]. They predominantly affect young men with high-energy injuries and older women with low-energy injuries [6].

Syndesmotic injuries are associated with more than 20% of ankle fractures. If not correctly diagnosed and treated, these injuries can lead to chronic pain, dysfunction, and even osteoarthritis, often necessitating surgery. Timely and accurate intervention is crucial to restore function and avoid long-term issues [7].

Tibiofibular syndesmosis includes four key ligaments: the interosseous membrane, transverse ligament, anterior inferior tibiofibular ligament (AITFL), and posterior inferior tibiofibular ligament (PITFL) [7]. In addition to the tibiofibular syndesmotic ligaments, the anterior talofibular ligament (ATFL) and the deltoid ligament are critical stabilizers of the ankle joint. These ligaments play a pivotal role in maintaining the stability of the lower ankle complex [8]. Importantly, they are among the most frequently injured structures in ankle trauma. Damage to the ATFL and deltoid ligament can significantly compromise joint stability, leading to persistent dysfunction if not accurately diagnosed and appropriately managed. Therefore, their integrity must be carefully assessed when evaluating and treating ankle injuries [9].

Surgical options for syndesmotic injuries focus on re-establishing proper anatomical alignment and stability. Traditional syndesmotic screws provide rigid fixation but may require removal after 3–4 months of healing [10]. Suture button devices offer more flexibility, allowing controlled micromotion that supports healing and reduces risks related to screw breakage or removal [11]. In severe cases, combining screws and suture buttons can enhance stability. Bioabsorbable implants are a newer option that avoids a secondary removal surgery, although their long-term effectiveness is still under review [11,12]. When fractures are present alongside syndesmotic injuries, open reduction and internal fixation (ORIF) address both issues [13].

Successful outcomes depend on precise anatomical reduction, as errors can result in persistent pain and dysfunction. The fixation method and individual factors such as age, activity level, and overall health also play a vital role in recovery [11,12,14].

Previous reviews indicated no significant differences in functional outcomes between routine removal of syndesmotic screws in tibiofibular injuries and retention [15]. However, both approaches carry risks, including implant breakage, infection, or fibula–tibia separation. Routine removal was considered unnecessary without specific clinical indications, as it introduces additional risks and costs without proven benefits. However, the strength of the evidence was insufficient, highlighting the need for further research in this area [16].

The aim of this study was to evaluate the impact of routine syndesmosis fixation removal on functional outcomes, complication rates, and overall recovery, with the goal of determining its clinical efficacy and relevance.

## 2. Materials and Methods

### 2.1. Study Design

This retrospective observational study examined patients treated for ankle fractures at the Department of Orthopedics and Trauma between 1 January 2016 and 31 March 2024. Data were extracted from the electronic patient database, identifying individuals who underwent surgical treatment for malleolar ankle fractures using a designated operation code. Surgical treatment decisions were made by attending trauma surgeons. Written informed consent was obtained from all participants, and the study protocol received approval from the Bioethical Commission of the Medical University of Łódź (RNN/138/24/KE). All procedures adhered to the ethical standards of the 1975 Declaration of Helsinki, as revised in 2013. The study was conducted according to STROBE guidelines for observational studies.

### 2.2. Patient and Public Involvement

Patients were not involved in the design or conduct of this study. However, those who expressed interest in the results will be informed upon publication. They will have access to the published article and a summary of the findings will be shared with them.

### 2.3. Setting

Baseline data included patient demographics (age, gender, smoking status), observation period, reoperations, and their indications. Fractures were classified according to the Weber classification or as isolated syndesmosis injuries, with further subgrouping based on reoperation status. Syndesmosis fixation methods (one three-cortical screw, one four-cortical screw, two screws, or a suture button) were analyzed using preoperative and postoperative imaging (computed tomography (CT) and X-ray scans of the affected ankle). Reoperations were defined as syndesmosis screw removal.

### 2.4. Inclusion and Exclusion Criteria

The study included patients aged 18 years or older who underwent surgical fixation for tibiofibular syndesmosis injuries. Exclusion criteria were age under 18 years, absence of syndesmosis injury, pilon fractures, initial surgery performed elsewhere, or a follow-up period shorter than eight weeks.

### 2.5. Surgical Technique

Fractures were managed using ORIF with specialized metal plates. Four syndesmosis fixation techniques were recorded: one three-cortical screw, one four-cortical screw, two screws, or a suture button. Intraoperative syndesmosis stability was assessed using the Cotton test (syndesmosis widening with lateral fibula traction) and fluoroscopic imaging [17]. Postoperative immobilization lasted 4–6 weeks, depending on whether the injury involved a fracture or isolated syndesmotic injury.

### 2.6. Study Group

A total of 407 patients underwent surgical tibiofibular syndesmosis stabilization between 2016 and 2024. After applying the exclusion criterion of a minimum 8-week follow-up, 124 patients were excluded due to insufficient follow-up duration. This left 283 eligible patients who were subsequently contacted for final outcome assessment. Of these, 170 patients responded and completed the evaluation using the Olerud–Molander Ankle Score (OMAS), a validated functional scale for assessing ankle joint performance. Based on their reoperation status, the respondents were categorized into three groups:

- Group 1: Syndesmosis stabilization was not removed.
- Group 2: Stabilization was routinely removed.
- Group 3: Stabilization removal was performed for specific indications, including infection, stabilization failure, persistent pain exceeding six months postsurgery, and nerve entrapment (reported in one patient).

Complications and subsequent hospitalizations associated with these interventions were recorded. Reported complications included infections and prolonged pain. All patients followed a standardized rehabilitation protocol (6 weeks’ immobilization, then progressive loading and physical therapy). Surgery timing was not significantly variable as procedures were performed within standard acute fracture management timelines.

### 2.7. Patient Outcomes Assessment

Patient-reported outcomes were evaluated using the OMAS, a standardized tool designed to measure ankle function following injury or surgery. The scale assesses functional abilities in daily activities, including walking, running, stair climbing, and squatting. It comprises nine items, each contributing to a total score ranging from 0 to 100, with higher scores reflecting better ankle function. OMAS measures pain, stiffness, swelling, and overall activity level, providing a comprehensive assessment of both physical limitations and subjective symptoms. The tool is widely applied in clinical and research settings for monitoring recovery and evaluating the effectiveness of treatments [18,19].

### 2.8. Statistical Analysis

Qualitative variables were presented as numbers with corresponding percentages. Continuous variables, which did not follow a normal distribution, were expressed as medians with interquartile ranges (25th–75th percentile). Normality was assessed using the Shapiro–Wilk test. Differences between groups were analyzed using non-parametric tests: the Mann–Whitney U test for two groups and the Kruskal–Wallis ANOVA for more than two groups. Correlations were evaluated using Spearman’s rank correlation coefficient. Statistical significance was set at *p* < 0.05, with *p*-values provided in the results. Statistically significant differences between groups were illustrated graphically. All analyses were performed using Statistica 13.1 software (Tibco, Palo Alto, CA, USA).

## 3. Results

### 3.1. Participants

The study included 170 patients, comprising 80 females and 90 males. Detailed patient characteristics are available in Table 1 and Table 2.

### 3.2. Complications and Hospitalization in the Routinely Reoperated Group—Univariable Analysis

Out of 52 patients who underwent routine reoperation, 5 (9.62%) experienced complications, with 2 (3.85%) requiring hospitalization. Among 22 patients reoperated due to specific indications, 2 (9.09%) had complications, but none required hospitalization. Out of 96 patients who did not undergo reoperation, 2 (2.08%) experienced complications, with none requiring hospitalization. Patients who underwent reoperation experienced a statistically higher number of complications, regardless of whether the reoperation was performed due to specific indications or resulted from the routine removal of the syndesmosis fixation (Table 3). The above analysis thus supports avoiding reoperations in patients, especially when there are no clear indications, as additional surgical intervention significantly increases the risk of postoperative complications.

### 3.3. OMAS Scale and Reoperation

No significant differences were observed in the OMASs between the groups (“indication-based reoperation”, “routine reoperation”, and “non-reoperated”), *p* = 0.646. OMAS comparison results should be interpreted carefully, considering follow-up differences.

### 3.4. OMAS Scale and Fracture Type

No significant differences were observed in the OMASs between the groups (Weber C, Weber B, synostosis), *p* = 0.782.

### 3.5. OMAS Scale and Fixation Type

No significant differences were observed in the OMASs between the groups (Endobutton, 1 three-cortical screw, 1 four-cortical screw, 2 screws), *p* = 0.280.

### 3.6. OMAS Scale and Age

**Figure 1:**
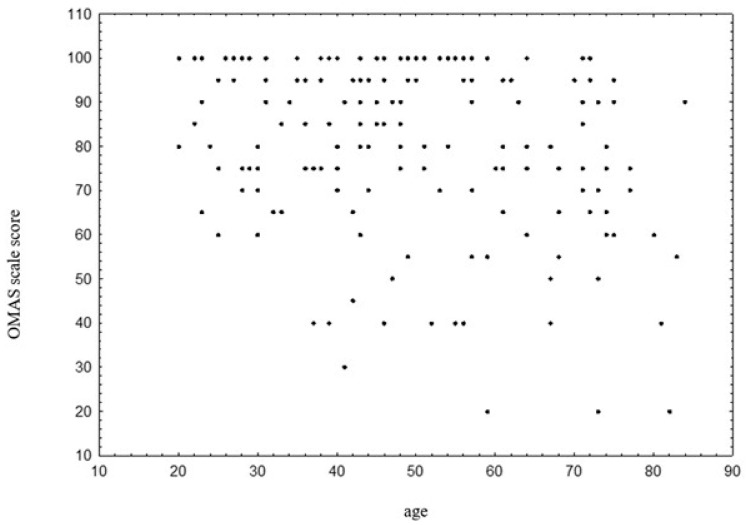
Correlation between age and OMASs. Abbreviations: OMAS—Olerud–Molander Ankle Score.

There was a statistically significant negative correlation between age and OMASs, *p* < 0.001, r = −0.296. Older patients tend to achieve lower scores on the OMAS scale (Figure 1).

### 3.7. OMAS Scale and Gender

**Figure 2:**
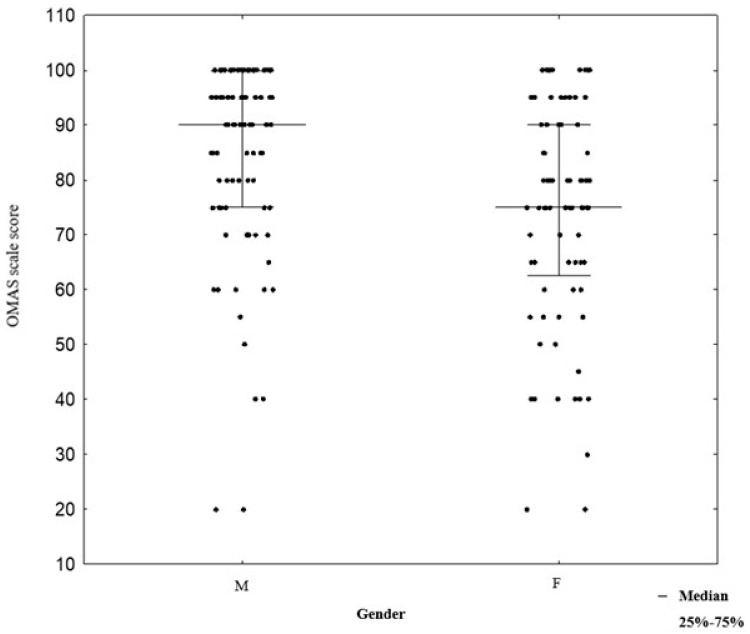
Correlation between gender and OMASs. Abbreviations: F—Female; M—Male; OMAS—Olerud–Molander Ankle Score.

In the male group, OMASs were higher (median 90, IQR (interquartile range) 75–100) compared to the female group (median 75, IQR 62.5–90), with the difference being statistically significant, *p* < 0.001 (Figure 2).

## 4. Discussion

The findings of this study indicate that routine removal of syndesmosis fixation does not offer significant functional benefits as measured by OMAS. Regardless of whether the fixation was retained, removed routinely, or removed based on specific clinical indications, the functional outcomes remained comparable across all patient groups [20,21]. These results align with previous studies suggesting that routine removal of syndesmotic screws may not be necessary in the absence of clinical symptoms or complications [22,23].

Notably, this study included one of the largest patient groups compared to previous similar studies and emphasized the relatively long follow-up period and comprehensive functional assessment using OMAS, further strengthening the validity of the findings.

Pogliacomi et al. [24] investigated the necessity and timing of syndesmotic screw removal after surgery for Weber type B and C ankle fractures, focusing on functional and radiological outcomes. The study included 90 patients who underwent ORIF with syndesmotic screw stabilization, divided into two groups: screw removed (Group 1) and screw retained (Group 2). Functional outcomes were assessed one year postsurgery using the OMAS and American Orthopaedic Foot & Ankle Society (AOFAS) scores, while radiological outcomes measured tibiofibular clear space immediately postsurgery and after one year. No significant differences were found in functional scores: Group 1 had a mean OMAS of 95 and AOFAS score of 94, compared to Group 2 with an OMAS of 92.5 and AOFAS score of 99 (*p* > 0.05). Tibiofibular clear space remained stable in both groups, and screw rupture, occurring in 29% of Group 2, had no adverse effects. The study highlighted that routine screw removal is unnecessary as it does not enhance long-term outcomes and carries surgical risks. If removal is chosen, proper timing is essential to ensure soft tissue healing, emphasizing individualized postoperative care [24].

Sanda et al. [25] assessed the impact of syndesmotic screw removal on quality of life, mobility, and daily activities in 144 patients treated for distal tibiofibular diastasis. Of these, 93 had screws removed, while 51 retained them. Data were collected two months postsurgery using standardized questionnaires, including the SF-36 Health Survey (SF-36), a short version of the World Health Organization Quality of Life assessment (WHOQOL-BREF), and Hospital Anxiety and Depression Scale (HADS). Screw removal was linked to higher mobility satisfaction (7.8 vs. 6.7; *p* = 0.018), improved daily living scores (8.1 vs. 6.5; *p* < 0.001), lower pain levels (5.3 vs. 6.8; *p* = 0.003), and reduced anxiety (5.8 vs. 7.3; *p* = 0.006). However, no significant differences were found in overall quality of life, depression scores, or patient willingness to recommend the approach. The study underscores specific benefits of screw removal, including better mobility, less pain, and lower anxiety, while noting no major impact on overall quality of life. It underscores the importance of personalized decision making to weigh benefits against risks such as infection or loss of reduction, providing valuable guidance for orthopedic care [25].

Desouky et al. [26] systematically reviewed eleven studies, including two randomized controlled trials (RCTs), to compare outcomes of syndesmotic screw removal versus retention in unstable ankle fractures. Functional outcomes, assessed with OMAS, AOFAS, and visual analog scale (VAS) scores, showed no significant differences between groups. Fractured screws were associated with better functional scores, suggesting they may not require removal. Routine removal posed risks, such as infection (up to 9.2%), while retained screws increased breakage and osteolysis without affecting outcomes [26].

A notable observation of the study was the increased rate of complications in reoperated patients, whether the reoperation was routine or indication-based, compared to those who did not undergo reoperation (9% vs. 2%). This emphasizes the inherent risks associated with additional surgical interventions, including infections and prolonged pain. Consequently, reoperation should be approached cautiously and reserved for patients with clear clinical indications such as infection, stabilization failure, or persistent discomfort exceeding six months postsurgery [27,28].

Dingemans et al. [29] conducted a systematic review to assess the necessity of syndesmotic screw removal after surgical fixation of unstable ankle fractures. The review included eleven studies (two RCTs and nine cohort studies). The findings showed no significant functional differences, including OMAS and range of motion, between patients with screws removed and retained. Complications of removal included infection, while retained screws posed risks of loosening or breakage without affecting clinical outcomes [29].

Walley et al. [30] systematically reviewed nine studies, including one RCT and eight retrospective cohort studies, to evaluate the necessity and timing of syndesmotic screw removal after ankle fracture fixation. The review assessed clinical, functional, and radiographic outcomes. Most studies, including the RCT, reported no significant differences in functional outcomes (AOFAS and OMASs) or tibiofibular alignment between retained and removed screws. Complications included infections with removal, particularly without antibiotics, and screw breakage or loosening with retention, though these did not worsen clinical outcomes [30,31].

Khurana et al. [32] conducted a meta-analysis of seven studies involving 522 patients to evaluate the necessity of routine syndesmotic screw removal after ankle fracture fixation. The review compared functional outcomes, pain levels, and complications between retained and removed screws. Results showed no significant differences in functional outcomes (AOFAS mean difference: −1.84, *p* = 0.150) or pain levels (VAS mean difference: −0.48, *p* = 0.390) between groups. Complication rates were similar (14.9% for removal vs. 13.1% for retention, *p* = 0.631). Routine removal added financial and procedural burdens without clinical benefits [32].

In the study patient age emerged as a significant factor influencing recovery, with younger patients achieving better OMASs compared to older individuals. This finding underscores the importance of considering age-related factors in the rehabilitation process and tailoring postoperative care to optimize outcomes for older patients [33,34]

The lack of significant differences in functional outcomes across various fixation and fracture types further reinforces the notion that the fixation method may not play a decisive role in long-term recovery. This supports a more individualized approach to syndesmosis fixation and removal, focusing on the patient’s clinical presentation rather than adhering to routine protocols [35,36]

Egol et al. [37] analyzed data from 347 patients with unstable ankle fractures to compare outcomes between those requiring syndesmotic stabilization and those undergoing only malleolar fixation. The study included patients with fractures classified using the Orthopaedic Trauma Association system. Syndesmotic injuries were confirmed via radiographic evidence or intraoperative stress tests, and postoperative care involved immobilization and physiotherapy with weight-bearing restrictions for 6–10 weeks. Of the patients, 79 (23%) required syndesmotic stabilization, more commonly in type C fractures (54%) than type B fractures (44%). At 6 and 12 months, the syndesmotic group had worse functional outcomes, including lower AOFAS and SMFA scores, and reported higher pain levels (*p* < 0.05). Syndesmotic screws broke in 13% of cases, but overall complication rates were similar between groups. The authors concluded that syndesmotic stabilization is linked to poorer functional outcomes at 12 months, highlighting the need to manage patient expectations [37].

## 5. Limitations

This study has several limitations that should be acknowledged. First, the retrospective design limits the ability to establish causal relationships and introduces potential biases related to patient selection and data collection. Second, the follow-up period varied among patients, which may have influenced the comparability of outcomes. This discrepancy is explained by clinical recovery patterns, with non-reoperated patients typically regaining full function earlier, thus exiting follow-up sooner compared to reoperated patients who required prolonged supervision due to complications or delayed recovery. Third, the response rate of 51.5% (170 out of 330 eligible patients) may introduce selection bias, as patients who responded could differ systematically from those who did not. Additionally, multivariate adjustment was not performed and should be addressed in future studies. Finally, the study was conducted at a single institution, which may limit the generalizability of the findings to other settings and populations.

## 6. Conclusions

Routine removal of syndesmosis fixation does not improve functional outcomes and is associated with an increased risk of complications. These findings emphasize the need to shift toward a more selective, patient-centered approach, prioritizing screw removal only in cases with clear clinical indications such as infection, fixation failure, or persistent pain. This study, based on one of the largest cohorts to date (n = 170), further confirms that routine reoperation offers no functional advantages, reinforcing the growing body of evidence against unnecessary surgical interventions. Future research should aim to validate these findings through prospective, multicenter trials, with careful adjustment for confounding factors such as patient age, fracture severity, and rehabilitation protocols. Moreover, special attention should be given to developing optimized rehabilitation strategies tailored to older patients, who may experience different recovery patterns and require individualized postoperative care.

## Figures and Tables

**Table 1:** Patient characteristics.

Variable		Reoperation Due to Indications N = 22 (%)	Routine Screw Removal N = 52 (%)	No Reoperation N = 96 (%)
Gender	Male	14 (63.64%)	26 (50.00%)	50 (52.08%)
	Female	8 (36.36%)	26 (50.00%)	46 (47.92%)
Diabetes	No	20 (90.91%)	50 (96.15%)	87 (90.63%)
	Yes	2 (9.09%)	2 (3.85%)	9 (9.37%)
Nicotine	No	14 (63.64%)	43 (82.69%)	79 (82.29%)
	Yes	8 (36.36%)	9 (17.31%)	17 (17.71%)
Fracture type	Weber B	19 (86.36%)	43 (82.69%)	64 (66.67%)
	Weber C	3 (13.64%)	8 (15.38%)	26 (27.08%)
	Synostosis	0 (0.00%)	1 (1.92%)	6 (6.25%)
Synthesis type	1 three-cortical screw	12 (54.55%)	38 (73.08%)	48 (50.00%)
	1 four-cortical screw	3 (13.64%)	11 (21.15%)	19 (19.79%)
	2 screws	6 (27.27%)	3 (5.77%)	16 (16.67%)
	Endobutton	1 (4.55%)	0 (0.00%)	13 (13.54%)

**Table 2:** Age, Olerud–Molander Ankle Score and follow-up time of the patients.

Variable	Reoperation Due to Indications Median (25–75%)	Routine Screw Removal Median (25–75%)	No Reoperation Median (25–75%)
Age	52 (42–57)	48 (39–69.5)	44 (34.5–61)
OMAS	80 (65 -100)	80 (70–95)	85 (72.5–95)
Follow-up time (months)	10 (8–17)	12 (6–19)	5 (3–8)

Abbreviations: OMAS—Olerud–Molander Ankle Score.

**Table 3:** Incidence of Complications Based on Reoperation.

	Reoperation	No Reoperation	*p*-Value
No Complications	67 (90.54%)	94 (97.92%)	0.042
Complications	7 (9.46%)	2 (2.08%)	

## Data Availability

The data presented in this study are available on request from the corresponding author (BGW).

## Abbreviations

The following abbreviations are used in this manuscript:

AITFL	anterior inferior tibiofibular ligament
AOFAS	American Orthopaedic Foot & Ankle Society
CT	computed tomography
HADS	Hospital Anxiety and Depression Scale
OMAS	Olerud–Molander Ankle Score
ORIF	open reduction and internal fixation
PITFL	posterior inferior tibiofibular ligament
PROMs	patient-reported outcomes
RCT	randomized controlled trial
SF-36	SF-36 Health Survey
SMFA	Short Musculoskeletal Function Assessment
VAS	visual analog scale
WHOQOL-BREF	short version of the World Health Organization Quality of Life
